# Pain in People with Multiple Sclerosis: Associations with Modifiable Lifestyle Factors, Fatigue, Depression, Anxiety, and Mental Health Quality of Life

**DOI:** 10.3389/fneur.2017.00461

**Published:** 2017-09-05

**Authors:** Claudia H. Marck, Alysha M. De Livera, Tracey J. Weiland, Pia L. Jelinek, Sandra L. Neate, Chelsea R. Brown, Keryn L. Taylor, Fary Khan, George A. Jelinek

**Affiliations:** ^1^Neuroepidemiology Unit, Melbourne School of Population and Global Health, The University of Melbourne, Melbourne, VIC, Australia; ^2^Biostatistics Unit, Melbourne School of Population and Global Health, The University of Melbourne, Melbourne, VIC, Australia; ^3^Sir Charles Gairdner Hospital, Nedlands, WA, Australia; ^4^Australian Rehabilitation Research Centre, Royal Melbourne Hospital, Department of Medicine, The University of Melbourne, Melbourne, VIC, Australia

**Keywords:** multiple sclerosis, pain, symptoms, lifestyle medicine, health outcome, disability

## Abstract

**Background:**

People with multiple sclerosis (MS) often experience pain, which can interfere with mobility, employment, and quality of life (QOL).

**Methods:**

This cross-sectional study explored associations between pain, demographic, disease, and modifiable lifestyle factors in an international sample of people with MS recruited online.

**Results:**

Substantial pain, of moderate/severe intensity and interfering at least moderately with work/household or enjoyment of life in the past 4 weeks, was reported by 682/2,362 (28.9%). Substantial pain was associated with fatigue (odds ratio (OR): 6.7, 95% confidence interval (CI): 4.9,9.3), depression (OR:4.0, 95% CI:3.2,5.1), anxiety (OR:2.4, 95% CI:1.9,2.9), and lower mental health QOL (Mean Difference: −14.7, 95% CI:−16.6,−12.8). Regression analyses showed that smoking (OR: 2.0, 95% CI:1.35,2.87) and obesity (OR:2.1, 95% CI: 1.5,2.8), moderate alcohol use (OR: 0.7, 95% CI:0.5,0.9), moderate (OR 0.7, 95% CI: 0.55,0.98) or high (OR 0.6, 95% CI: 0.4,0.8) physical activity level, and healthy diet (OR 0.8, 95% CI: 0.75,0.95, per 10 points) were associated with substantial pain.

**Conclusion:**

Our results show clear associations with modifiable lifestyle factors and substantial pain in MS. These factors are already considered in the prevention and management of pain in other populations but have not previously been considered in MS. Conversely, pain and associated common MS comorbidities, such as depression, anxiety, and fatigue, may hamper efforts to start or maintain healthy behaviors. Strategies to overcome these barriers need to be considered. Further research should clarify the direction of these associations.

## Introduction

Multiple sclerosis (MS) is a neurological disorder with variable disease course and diverse symptomatology. Demyelination in the central nervous system (CNS) causes symptoms such as fatigue, weakness and impairment in mobility, sensation, and vision.

Pain, including headaches, lower back pain, neuropathic pain and painful spasms, is a common symptom in people with MS (PwMS), with a wide prevalence range between 30 and 85% (1–4). In the general population, prevalence estimates of 20–30% are commonly reported (5, 6), although prevalence estimates from older populations report up to 72% (7). However, definitions and sample characteristics vary between studies, making it difficult to directly compare prevalence and severity of pain. One case–control study in PwMS found a similar prevalence of pain to controls [Odds Ratio (OR) 0.99–1.13], but showed that PwMS had more severe pain than controls, comparable to people with arthritis (8). Another study showed that both pain severity and interference was greater in PwMS than controls, but these differences were only significant in some, mostly older age groups (9). PwMS more often require pharmacological treatment (10) and pain can significantly interfere with daily activities (11) and employment (12) and affect quality of life (QOL) (13–15).

Damage in the central or peripheral nervous system, inflammatory, and musculoskeletal mechanisms, such as immobilization of parts of the body, may all contribute to pain (4, 16). Exogenous contributors include MS drugs, such as interferon beta, dimethyl-fumarate and long-term steroid treatment (4, 17), although no associations between MS drugs and pain, were found in a large North American study (14).

In MS, pain seems to be part of a complex interplay between other common symptoms or comorbidities, such as fatigue, anxiety, and depression (18, 19), although at least one study suggests the associations between anxiety, depression, and pain is present only in women (10). Bio-psycho-social models, recognizing psychological and environmental factors, have recently been proposed to disentangle the causal pathway between these factors. Different outcome measures have been assessed using comprehensive models, including a recent study showing that pain affects participation in life through fatigue and physical functioning (20). Modifiable lifestyle factors associated with MS onset and progression, such as vitamin D, smoking, stress, diet, physical activity, and body mass index (BMI) (21), have, however, not been studied in relation to pain to great extent.

Our study aimed to determine the associations between substantial pain and modifiable lifestyle factors and to explore the relationship between substantial pain, depression, anxiety, and fatigue in a large international sample of PwMS.

## Materials and Methods

### Participants and Data Collection

The methodology of the *H*ealth *O*utcomes and *L*ifestyle *I*n a *S*ample of people with *M*ultiple sclerosis study has previously been described (22). Participants over 18 years who reported a diagnosis of MS by a medical doctor were recruited *via* online platforms, including social media, websites, and forums that engaged PwMS, most of which had a health and lifestyle focus in 2012. Participants reporting a possible diagnosis of MS or Clinically Isolated Syndrome were not included in this analysis. The web-based tool, SurveyMonkey^®^, was used to collect responses. As the survey was in English only, non-English speaking participants were not included.

### Ethics, Consent, and Permissions

The Health Sciences Human Ethics Sub-Committee at the University of Melbourne provided ethical approval for the study (Ethics ID: 1545102). Individuals were provided with participant information and could not enter the survey until they confirmed they were 18 years or older and consented to participate.

### Data Collected and Tools Used

The online survey collected data in 2012, used validated tools where possible, and took approximately 40 min to complete. Specific to this study were items exploring demographic variables, morbidity indicators, and modifiable lifestyle factors. Demographic factors were collected using researcher-devised items and included age and gender.

### Substantial Pain

Items regarding pain were part of the Multiple Sclerosis Quality Of Life (MSQOL-54) instrument and included three items regarding intensity of pain; interference of pain with work or household activities; and interference with enjoyment of life. Almost a quarter (24.7%) reported no pain in the 4 weeks prior to survey, while 44.3% reported no interference with normal work or household activities and 43.7% reported no interference with enjoyment of life (see Table S1 in Supplementary Material). As our aim was to assess substantial pain, we defined this as having moderate, severe, or very severe pain, which interfered moderately, quite a bit or extremely with normal work/household activities and/or enjoyment of life in the past 4 weeks. Substantial pain was experienced by 682 participants (28.9%). The results section describes the characteristics of the sample with sufficient data to determine the prevalence of substantial pain.

### Morbidity Indicators

Mental health-related QOL was also assessed by the widely used MSQOL-54, giving rise to the mental health QOL scores, as well as several other scales not included in these analyses. Level of gait disability was assessed using the Patient Determined Disease Steps, a validated self-reported surrogate tool to the Expanded Disability Status Scale, with ordinal scores from 0 (no disability) to 8 (bed-bound). Researcher-devised items assessed self-reported doctor-diagnosed relapse rate over the previous 12 months, type of MS currently diagnosed, and years since diagnosis. The Patient Health Questionnaire depression module short version (PHQ-2) was used to screen for depression risk; a score of 3 or more indicated a positive screen for depression. The fatigue severity scale, with a cutoff of 4 or more, was used as an indicator of clinically significant fatigue. A researcher-devised item assessed current anxiety. A list of 24 disease-modifying drugs (DMDs) was provided and participants were categorized as either currently using a DMD or not.

### Modifiable Lifestyle Factors

Height and weight information allowed calculation of BMI according to the World Health Organization criteria (<20 = underweight, 20–25 = normal weight, 25–30 = overweight, and >30 = obese). Dietary habits were assessed with the modified Dietary Habits Questionnaire on a scale of 0–100. The International Physical Activity Questionnaire was included to assess level of regular physical activity (low, moderate, and high). Omega-3 and vitamin D supplementation (yes/no), meditation practice (never, ≤once a week, >once a week), level of alcohol consumption [low (<15 g/week), moderate/high (<15 g/week)], and current smoking status (current/previous/never) were assessed using researcher-devised items. All data were self-reported.

### Statistical Analysis

Categorical data are presented as percentages with frequency. Continuous data are presented as mean and SD, and skewed data are presented as median with interquartile range (25th–75th percentile). We used univariable and multivariable logistic regression models to investigate the associations between substantial pain and a range of modifiable lifestyle factors adjusting for confounders identified *a priori* based on our subject-matter knowledge (18, 20). This model included all modifiable lifestyle variables and age, gender, years since diagnosis, and level of disability. In addition, we explored the associations between pain and fatigue, depression, anxiety, and mental health QOL using separate multivariable logistic regression models, each adjusted for confounders age, gender, disease duration and type of MS. Data were analyzed using Stata, V12 (StataCorp, College Station, TX, USA) and R (R Foundation for Statistical Computing, Vienna, Austria).

## Results

The sample characteristics of the 2,362 participants who completed data items for the substantial pain variable are shown in Table 1, with a full list of country of residence available in a previous publication (22). Unadjusted univariable analysis showed that several socio-demographic variables (Table 1), and health outcomes (Table 2) were associated with substantial pain.

**Table 1:** Sample characteristics and their unadjusted associations with substantial pain obtained using univariable regression models.

		*N* (%) or mean [95% confidence interval (CI)]	Unadjusted odds ratio (95% CI)
Age	*N* = 2,295	45.5 (45.1–46.0)	1.02 (1.01–1.03)

Gender	Male	407 (17.8)	Reference
	Female	1,885 (82.2)	1.81 (1.39–2.35)

Years since diagnosis	First quartile (<4)	684 (29.1)	Reference
	Second quartile (4–7)	529 (22.5)	1.26 (0.97–1.63)
	Third quartile (7–13)	575 (24.5)	1.63 (1.27–2.09)
	Fourth quartile (>13)	564 (24.0)	1.55 (1.21–1.99)

Type of multiple sclerosis (MS)	Relapsing remitting	1,448 (61.5)	Reference
	Benign	97 (4.1)	0.32 (0.16–0.62)
	Primary progressive	170 (7.2)	1.39 (0.99–1.95)
	Secondary progressive	267 (11.4)	1.99 (1.52–2.61)
	Progressive relapsing	47 (2.0)	3.73 (2.07–6.72)
	Other	324 (13.8)	1.05 (0.80–1.37)

Relapse in previous 12 months (relapsing remitting MS only)	Yes	654 (46.3)	2.46 (2.03–2.97)
	No	759 (53.7)	Reference

Disability level	Normal to some disability	1,263 (55.2)	Reference
	Gait/cane disability	787 (34.4)	3.42 (2.79–4.19)
	Major mobility support	240 (10.5)	4.01 (2.99–5.38)

DMD use	Yes	1,133 (50.0)	1.12 (0.94–1.35)
	No	1,132 (50.0)	Reference

Pain medication	No pain medication	1,178 (52.0)	Reference
	Over the counter or herbal pain medication only	611 (27.0)	3.39 (2.66–4.33)
	Prescription pain medication^a^	476 (21.0)	13.39 (10.36–17.32)

*DMD, disease-modifying drug*.

*^a^Some people taking prescription medication were also taking over the counter or herbal pain medication*.

**Table 2:** Adjusted associations of health outcomes with substantial pain.^a^

		Number (%) or mean (SD)	Adjusted odds ratio (95% confidence interval)
Depression screen	Negative	1,790 (80.8)	Reference
	Positive	425 (19.2)	4.03 (3.17, 5.14)
Significant fatigue	Negative	733 (34.5)	Reference
	Positive	1,395 (65.6)	6.74 (4.89, 9.31)
Anxiety	No	1,676 (71.0)	Reference
	Yes	684 (29.0)	2.36 (1.91, 2.92)
Mental health QOL	*N* = 2,272	66.6 (65.7–67.5)	−14.7 (−16.6 to −12.8)

*^a^Adjusted for age, gender, duration of MS and level of disability. QOL, quality of life*.

In addition, we explored the association between several modifiable lifestyle factors as exposures and substantial pain as the outcome in a multivariable regression model adjusted for age, gender, duration of MS and level of disability. Table S2 in Supplementary Material shows the lifestyle factors for the sample. Participants who smoked (compared with those who never smoked) or were obese (compared with those with normal BMI) were twice as likely to report substantial pain. Those with moderate or high alcohol use (compared with low) and those with moderate levels of physical activity (compared with low) were 1.4–1.5 times less likely to report substantial pain, while those reporting high physical activity were 1.7 times less likely to report substantial pain; every 10 point increase in healthy diet score was associated with 1.2 times lower odds of substantial pain (Table 3).

**Table 3:** Associations between modifiable lifestyle factors and substantial pain, obtained using multivariable regression modeling.^a^

		Odds ratio	95% confidence interval		*p*-Value
Smoking status	Never	Reference			
	Previous	1.00	0.77	1.30	0.984
	Current	1.97	1.35	2.87	<0.001

Alcohol use	Low	Reference			
	Moderate/high	0.67	0.52	0.87	0.002

Physical activity	Low active	Reference			
	Moderate active	0.73	0.55	0.98	0.037
	High active	0.58	0.42	0.80	0.001

Daily vitamin D supplementation	None	Reference			
	1–5,000 IU	0.90	0.65	1.25	0.538
	>5,000 IU	0.77	0.51	1.16	0.208

Omega-3 supplementation	None	Reference			
	Flaxseed	0.87	0.53	1.42	0.577
	Fish oil	0.89	0.67	1.20	0.451
	Both fish and flaxseed oil	0.79	0.53	1.19	0.263

Meditation frequency	Never	Reference			
	Yes	1.27	0.98	1.64	0.065

Body mass index	Normal weight	Reference			
	Underweight	1.07	0.57	1.98	0.842
	Overweight	1.33	0.99	1.81	0.062
	Obese	2.06	1.51	2.81	<0.001

Dietary score (per 10 points)		0.84	0.75	0.95	0.004

DMD use	No	Reference			
	Yes	1.07	0.83	1.37	0.589

Pain medication use	No	Reference			
	Prescription*	8.34	6.13	11.34	<0.001
	Over the counter or herbal only	2.67	2.00	3.57	<0.001

*Complete case analysis consisted of 80% of the data, *N* = 1,955. DMD, disease-modifying drug. LR χ^2^(23) = 630.4, *p* < 0.001, Log likelihood = −855.7, Pseudo *R*^2^ = 0.27*.

*^a^Model adjusted for age, gender, years since diagnosis and disability level*.

*Some people taking prescription medication were also taking over the counter or herbal pain medication.

## Discussion

Pain is common in PwMS and contributes significantly to disability (11, 12) and worse QOL (13, 14). Our results showed that those with substantial pain were more likely to be diagnosed longer ago and had a higher level of disability and more relapses. Most studies report associations between the likelihood of experiencing pain and pain intensity with longer disease duration, more progressive forms of MS and level of disability (14, 23, 24), while some do not (3, 25, 26). Inconsistent findings of associations between pain and relapses have also been reported, with some showing associations of pain with higher relapse rate (23), while others report increased relapses associated with lower prevalence of pain (26). In univariable analyses, we found a reduced risk of pain in people with relapsing remitting MS (RRMS), older age and female gender, in line with others (14). Age, gender and years since diagnosis were, however, no longer significantly associated with substantial pain in a multivariable regression adjusting for modifiable lifestyle factors, while disability level was still strongly related to pain.

Reduced mental health QOL, fatigue, depression, and anxiety had substantial adjusted associations with substantial pain, in line with previous literature (2, 15, 18, 20, 27). Pain is a multi-factorial symptom, with clear physical correlation in PwMS with CNS demyelination and degeneration (28), but also contributed to by psycho-social factors such as personality, depression, anxiety, and comorbid conditions, such as fatigue (18). The interplay between these factors determines the perception of pain. The presence of pain in its own right may, however, also lead to some of these comorbidities, as may poorer lifestyle behaviors, which in turn may be maintained due to the presence of pain (e.g., physical inactivity, and smoking).

Our study identified important associations between the experience of substantial pain and modifiable lifestyle risk factors. Current smokers were twice as likely to report substantial pain in our study, in line with other studies (29). Another study showed that current smoking increased the likelihood of pain in those reporting depression (30). The association between smoking and pain has been summarized as a positive feedback loop where smoking may be a risk factor for pain, while pain is a powerful motivator to continue smoking due to anti-nociceptive effects (31). This feedback loop may be especially hard to break for people experiencing depression or anxiety who may have increased pain sensitivity and negative affect when attempting smoking cessation (32). Clinicians should be aware of this interplay when managing MS symptoms. Promoting smoking cessation as part of a secondary and tertiary management strategy is, however, vital since smoking has been shown to play a significant role in disease progression (33), although this may be more difficult in people experiencing pain or depression.

Similarly, we showed a significant association between more physical activity and lower odds of experiencing substantial pain, in line with previous literature (34). While people may be less likely to engage in physical activity due to pain, there may well be a role for physical activity in pain management in PwMS (35). Physical activity is known to increase pain threshold and tolerance (36). In a study of older adults, those with chronic pain had lower levels of physical activity, and fear-avoidance believes played an important role in predicting future physical activity levels, over pain characteristics (37). Others have shown that psycho-social factors, including negative beliefs about pain and avoidance of activity are likely to influence pain severity and interference (38). Promoting physical activity among PwMS is of importance given the wide-ranging benefits of exercise in potentially modifying the course of the disease (39) and reducing fatigue and depression (40–42); however, consideration of psycho-social barriers to increasing physical activity is essential.

Poorer diet and obesity were associated with higher odds for substantial pain in our study. Little is published about this association in humans but there is some evidence from animal studies that a high-fat diet can contribute to pain (43, 44), and it is plausible that poor diet contributes to systemic inflammation and increased pain sensitivity (45). Poor diet may lead to becoming overweight or obese which in itself is associated with increased likelihood of pain (30, 46). Weight loss can improve pain symptoms (47, 48), although pain can also be a barrier to weight loss (49). A population-based cohort study showed that being overweight or obese was associated with reporting future pain, while pain increased the odds for future obesity, with smoking confounding this relationship (19). As with diet, chronic inflammation is likely implicated in this interplay between physical activity, weight, and pain (50). Obesity has become a focus in secondary prevention in MS, with clear associations with poorer health outcomes (51). Our findings suggest that moderate alcohol use need not be considered negative for PwMS, in line with previous research (52).

Based on our data, it is unclear whether these modifiable lifestyle factors directly contribute to development of substantial pain in MS, or indirectly through fatigue, anxiety, and/or depression; or whether pain is contributing to fatigue, depression and poorer lifestyle behaviors. Future studies could further investigate these associations by attempting to clarify temporality through longitudinal data, and investigation mediating and moderating factors involved. Future studies should also record the type and potentially the cause of pain, to further understand the underlying mechanism.

Depression, smoking, physical activity, and being overweight have previously been reported as modifiable risk factors to be considered in the prevention and management of pain in other populations (30, 53). It is likely that a comprehensive secondary and tertiary prevention strategy for pain (and health outcomes in general) in PwMS would include advice about lifestyle factors such as diet, weight, physical activity, and smoking (21). Rehabilitation services often address lifestyle factors that can minimize the impact of pain in PwMS (54). However, pain, psycho-social factors, and common symptoms or comorbidities such as depression, anxiety, and fatigue, may hamper efforts to take up or maintain healthy behaviors. Holistic strategies to overcome these barriers and break the feedback loop need to be considered.

### Strengths and Limitations

To our knowledge, this is the first study to report on a range of modifiable lifestyle factors in relation to MS-associated pain. This study is international in scope with a large sample size, using multivariable regression, including a wide range of relevant variables. However, sleep disturbance, previously indicated in the relation between depression and pain, was not measured in our sample (18). We also did not record the type of pain medication (other than whether they were prescription or over the counter pain medication) or the location of pain. All data were self-reported and, although previously validated tools were used where possible, were unable to be verified. Substantial pain was determined by categorizing participants in two groups depending on their responses to items on the pain scale of the MSQOL-54; this method has not previously been used or validated, but does allow for future studies to directly compare data with ours. High alcohol use was very rare among our participants and they were grouped with moderate alcohol users. Due to participation bias, this sample may not be generalizable to all PwMS, despite the variety of backgrounds of participants and size of the sample. Finally, our study is cross-sectional; therefore, we cannot assume causal or temporal relationships. The assessment of the complex interplay between the variables (e.g., the direct and indirect contribution of the factors described here to substantial pain) was beyond the scope of this paper. We used complete case analysis, restricted to available data. Table S3 in Supplementary Material summarizes the distribution of the variables of the data used and not used in the analysis presented in Table 3, indicating that the difference between the two datasets is minimal.

## Conclusion

Pain is common in PwMS, with substantial pain present in over a quarter of our study population. Fatigue, depression, reduced mental health QOL, and anxiety were strongly associated with the presence of substantial pain. For the first time, we showed that tobacco smoking, obesity, lack of exercise, and poor diet were also associated with pain using multivariable analyses. These modifiable factors have previously been suggested to be targets for management of pain in other populations and in the secondary prevention of MS. Further research is needed to disentangle the complex interplay between these lifestyle factors, symptoms, and comorbidities, which may contribute to pain, or may be caused or maintained by the presence of pain.

## Ethics Statement

The Health Sciences Human Ethics Sub-Committee at the University of Melbourne provided ethical approval for the study (Ethics ID: 1545102). Individuals were provided with participant information and could not enter the survey until they confirmed they were 18 years or older and consented to participate.

## Author Contributions

CM, TW, and GJ were involved in conceiving the study. CM was involved in data collection, and CM, CB, and AL contributed to cleaning and analyzing the data. CM drafted the manuscript with the help of PJ and FK. All authors edited and contributed to the final version and approved the manuscript.

## Conflict of Interest Statement

GJ receives royalties for his books Overcoming Multiple Sclerosis and Recovering from Multiple Sclerosis. GJ, SN, and KT have received remuneration for conducting lifestyle educational interventions for PwMS. All other authors declare no conflict of interest.
