# Supplementary material for: Pain in People with Multiple Sclerosis: Associations with Modifiable Lifestyle Factors, Fatigue, Depression, Anxiety, and Mental Health Quality of Life
Source: Front Neurol. 2017 Sep 5;8:461. doi: 10.3389/fneur.2017.00461 (PMC5591834; doi:10.3389/fneur.2017.00461)
Supplement: Supplementary file 1 [file Table_1.DOCX]

Supplementary Table 1. Bodily pain and interference of pain in past four weeks

|  | Bodily pain | |  | Interference with normal work/household activities | | Interference with enjoyment of life | |
| --- | --- | --- | --- | --- | --- | --- | --- |
|  | N | % |  | N | % | N | % |
| None | 583 | 24.7 |  |  |  |  |  |
| Very mild | 563 | 23.8 | Not at all | 1048 | 44.3 | 1010 | 43.7 |
| Mild | 408 | 17.3 | A little bit | 633 | 26.8 | 667 | 28.9 |
| Moderate | 568 | 24.0 | Moderately | 362 | 15.3 | 336 | 14.6 |
| Severe | 206 | 8.7 | Quite a bit | 232 | 9.8 | 229 | 9.9 |
| Very severe | 37 | 1.6 | Extremely | 89 | 3.8 | 67 | 2.9 |
| Total | 2365 | 100 | Total | 2364 | 100 | 2309 | 100 |
