# Supplementary material for: Pain in People with Multiple Sclerosis: Associations with Modifiable Lifestyle Factors, Fatigue, Depression, Anxiety, and Mental Health Quality of Life
Source: Front Neurol. 2017 Sep 5;8:461. doi: 10.3389/fneur.2017.00461 (PMC5591834; doi:10.3389/fneur.2017.00461)
Supplement: Supplementary file 2 [file Table_2.DOCX]

Supplementary Table 2. Modifiable lifestyle factors

|  |  | N (%) or mean (95%CI) |
| --- | --- | --- |
| Smoking status | Never | 1099 (48.2) |
|  | Previous | 913 (40.1) |
|  | Current | 267 (11.7) |
| Alcohol use | Low | 1394 (61.6) |
|  | Moderate/high | 871 (38.5) |
| Physical activity | Low active | 920 (41.1) |
|  | Moderate active | 701 (31.6) |
|  | High active | 601 (27.1) |
| Daily vitamin D supplementation | None | 400 (18.3) |
|  | 1-5000 IU | 1338 (61.3) |
|  | >5000 IU | 446 (20.4) |
| Omega 3 supplementation | None | 804 (36.8) |
|  | Flaxseed | 203 (9.3) |
|  | Fish oil | 786 (35.9) |
|  | Both | 395 (18.1) |
| Fish consumption | Less than once a week | 654 (28.7) |
|  | 1-2 times per week | 919 (40.3) |
|  | >2 per week | 706 (31.0) |
| Meditation | Never | 1067 (47.8) |
|  | Yes | 1167 (52.2) |
| Body mass index | Normal weight | 1246 (53.5) |
|  | Underweight | 97 (4.2) |
|  | Overweight | 535 (23.0) |
|  | Obese | 452 (19.4) |
| Dietary score | N=2284 | 78.9 (78.4-79.4) |

Some variables had missing data, the denominator was adjusted accordingly.
