# Supplementary material for: Pain in People with Multiple Sclerosis: Associations with Modifiable Lifestyle Factors, Fatigue, Depression, Anxiety, and Mental Health Quality of Life
Source: Front Neurol. 2017 Sep 5;8:461. doi: 10.3389/fneur.2017.00461 (PMC5591834; doi:10.3389/fneur.2017.00461)
Supplement: Supplementary file 3 [file Table_3.DOCX]

Supplementary Table 3. The distribution of the variables of the data used (non-missing) and not used (missing) in the analysis presented in Table 3.

|  | Missing  N(%) or mean (sd) | | Non-Missing  N (%) or mean (sd) |
| --- | --- | --- | --- |
| Age* | 45.56 (10.06) | 45.52 (10.64) | |
| Years since diagnosis |  |  | |
| First quartile | 138(29.3%) | 568 (29.1%) | |
| Second quartile | 99 (21.1%) | 444 (22.8%) | |
| Third quartile | 107 (22.7%) | 482 (24.7%) | |
| Fourth quartile | 127 (26.9%) | 455 (23.4%) | |
| Gender |  |  | |
| Male | 68 (19.2%) | 339 (17.4%) | |
| Female | 286 (80.8%) | 1610 (82.6%) | |
| Disability level |  |  | |
| Normal to some | 178 (50.7%) | 1089 (55.9%) | |
| Gait/cane | 138 (39.3%) | 655 (33.6%) | |
| Major mobility | 35 (10%) | 205 (10.5%) | |

*mean (sd)
